# Redesigning care for older people to preserve physical and mental capacity: WHO guidelines on community-level interventions in integrated care

**DOI:** 10.1371/journal.pmed.1002948

**Published:** 2019-10-18

**Authors:** Jotheeswaran Amuthavalli Thiyagarajan, Islene Araujo de Carvalho, Juan Pablo Peña-Rosas, Shelly Chadha, Silvio Paolo Mariotti, Tarun Dua, Emiliano Albanese, Olivier Bruyère, Matteo Cesari, Alan Dangour, Amit Dias, Mariella Guerra, Jill Keeffe, Ngaire Kerse, Qurat ul Ain Khan, Chiung-ju Liu, Gudlavalleti V. S. Murthy, Serah Nyambura Ndegwa, Jean-Yves Reginster, Luis Miguel F. Gutiérrez Robledo, Kelly Tremblay, Jean Woo, Martin Prince, John R. Beard

**Affiliations:** 1 Department of Ageing and Life Course, World Health Organization, Geneva, Switzerland; 2 Department of Nutrition for Health and Development, World Health Organization, Geneva, Switzerland; 3 Department for Management of Noncommunicable Diseases, Disability, Violence and Injury Prevention, World Health Organization, Geneva, Switzerland; 4 Department of Mental Health and Substance Abuse, World Health Organization, Geneva, Switzerland; 5 World Health Organization Collaborating Center for Research and Training in Mental Health, University of Geneva, Geneva, Switzerland; 6 World Health Organization Collaborating Center for Public Health Aspects of Musculoskeletal Health and Aging, University of Liège, Liège, Belgium; 7 Geriatric Unit, Fondazione IRCCS Ca’ Granda–Ospedale Maggiore Policlinico, Università di Milano, Milan, Italy; 8 London School of Hygiene & Tropical Medicine, London, United Kingdom; 9 Goa Medical College, Goa, India; 10 Institute of Memory, Depression and Related Disorders, Lima, Peru; 11 World Health Organization Collaborating Centre for Prevention of Blindness, LV Prasad Eye Institute, Hyderabad, India; 12 University of Auckland, Auckland, New Zealand; 13 Aga Khan University Hospital, Karachi, Pakistan; 14 Indiana University, Indianapolis, Indiana, United States of America; 15 Indian Institute of Public Health, Hyderabad, Madhapur, India; 16 University of Nairobi, Nairobi, Kenya; 17 Institutos Nacionales de Salud de México, Mexico City, Mexico; 18 University of Washington, Seattle, United States of America; 19 The Chinese University of Hong Kong, Hong Kong, China; 20 Institute of Psychiatry, Psychology & Neuroscience, King’s College London, London, United Kingdom; 21 ARC Centre of Excellence on Population Ageing Research, University of New South Wales, Sydney, New South Wales, Australia

## Abstract

Islene Araujo de Carvalho and coauthors discuss the WHO guidelines on integrated care for older people.

**Summary points**

- Numerous underlying physiological changes occur with increasing age, and for older people, the risks of developing chronic diseases and care dependency increase.
- Health systems are often better designed to respond to episodic health needs than to the more complex and chronic health needs that tend to arise with increasing age, and reprioritization is required to meet the needs of ageing populations.
- Significant losses in capacity and care dependency in later life can be delayed or avoided if health interventions are introduced earlier in the process of functional decline.
- We discuss the recommendations of the WHO Integrated Care for Older People (ICOPE) Guidelines, which provide evidence-based recommendations for managing declines in intrinsic capacity, developed with the Grading of Recommendations Assessment, Development and Evaluation (GRADE) methodology.
- Implementation of evidence-based interventions requires community-based assessment of an individual’s needs, development and implementation of a care plan, provision of monitoring and referrals as needed, and supporting caregivers.

## Background

Today, for the first time in history most people can expect to live into their 60s and beyond [1]. In low- and middle-income countries, this is largely the result of large reductions in mortality at younger ages, particularly during childhood and childbirth, and from infectious diseases. In high-income countries, continuing increases in life expectancy are now mainly due to declining mortality among those who are older. Even in sub-Saharan Africa, which has the world’s youngest population structure, the number of people over 60 years of age is expected to increase over 3-fold, from 46 million in 2015 to 147 million in 2050 [2].

Ageing is associated with numerous underlying physiological changes and increased risk of experiencing more than one chronic condition at the same time (known as multimorbidity) [3]. The impact of multimorbidity on an older person’s capacity, healthcare utilization, and their costs of care is often significantly greater than might be expected from the summed effects of each condition [4]. These are not problems just for higher-income countries; in fact, the burden associated with chronic conditions in older age is generally far higher in low- and middle-income countries [5]. One consequence of these disease patterns is that population ageing will dramatically increase the number of people needing long-term care in countries at all levels of development [6]. At the same time, the number of younger people who might be available to provide care will fall, and the societal role of women, who have until now been the main care providers, is changing.

In 2015, the World Health Organization (WHO) articulated a new vision for healthy ageing in its *World Report on Ageing and Health* [7]. The report defines healthy ageing as “the process of developing and maintaining the functional ability that enables well-being.” This functional ability is determined by the intrinsic capacity of the individual, relevant environmental characteristics, and the interactions between them. Although actions at all levels of society are vital to foster healthy ageing, realigning health systems towards building and maintaining the intrinsic capacity of adults in the second half of life has been identified as an immediate priority [8].

Intrinsic capacity was defined as the composite of the physical and mental capacities of an individual [7]. Losses of intrinsic capacity in older age are frequently characterized by the manifestation of common problems, such as difficulties with hearing, seeing, memory, walking at usual pace, continence, and positive affect [8]. Yet, most healthcare professionals currently lack guidance or training to recognize and manage declines in physical and mental capacities in older age [9]. Moreover, based on the belief that there is no treatment available for their problems, older people may disengage from services, not adhere to treatment, and/or not attend primary healthcare clinics. However, research suggests that even in low-resourced healthcare settings, healthcare professionals can be trained to detect declines in physical and mental capacities (clinically expressed as impairments) and deliver effective interventions to prevent and delay progression [10]. Therefore, there is a pressing need to develop comprehensive evidence-based clinical guidelines and train health professionals to provide person-centered and integrated care for older people, and support informal caregivers.

The paper discusses the WHO Guidelines on Integrated Care for Older People (ICOPE) [11] published in 2017 and presents recommendations on community-level interventions to manage declines in intrinsic capacity.

## WHO methodology for the development of guidelines

The priority conditions discussed in the guidelines were selected because they relate to reductions in physical and mental capacities, as outlined in the WHO framework on healthy ageing [7], and are strong independent predictors of mortality and care dependence in older age [12].

The WHO recommendations are based on evidence from around 600 randomized controlled trials published up until 2015 and consideration of values, preferences, and feasibility issues from an international perspective [11]. These global evidence-informed recommendations were developed by WHO, following a rigorous guideline development methodology. The detailed methodology of WHO guideline development is described elsewhere [13]. A brief overview is presented in the Supporting information. A guideline development group (GDG) and an external peer review group were established to assist in the formulation of the recommendations. The GDG included a panel of academics and clinicians with multidisciplinary expertise on the conditions covered by the guidelines, and geriatricians and doctors specializing in the care of older people.

## WHO-ICOPE recommendations

The primary audience for the WHO-ICOPE guidelines on community-level interventions is healthcare providers working in primary and secondary care settings. While the recommendations are specific to the community setting, many are also applicable to healthcare facilities.

Because most of the conditions selected for these guidelines share the same underlying factors and determinant conditions, it may be possible to prevent or delay the onset of losses in intrinsic capacity through an integrated approach to modifying a set of predisposing factors. For example, highly intensive strength training is the key intervention necessary to prevent and reverse mobility impairments, but it also indirectly protects the brain against depression and cognitive decline and prevents falls. Nutrition enhances the effects of exercise and has a direct impact on increasing muscle mass and strength. It is therefore important to implement these guidelines using an older person–centered and integrated approach, and the recommendations sought to reflect this. The recommendations are considered under six overarching actions. All specific recommendations are presented in Box 1.

**Box 1. WHO-ICOPE guidelines recommendations**

***Improve musculoskeletal function, mobility, and vitality***

**Recommendation 1:** Multimodal exercise, including progressive strength resistance training and other exercise components (balance, flexibility, and aerobic training) should be recommended for older people with declining physical capacity, measured by gait speed, grip strength, and other physical performance measures (Quality of the evidence, moderate; Strength of the recommendation, strong).

**Recommendation 2:** Oral supplemental nutrition with dietary advice should be recommended for older people affected by undernutrition (Quality of the evidence, moderate; Strength of the recommendation, strong).

***Maintain sensory capacity***

**Recommendation 3:** Older people should receive routine screening for visual impairment in the primary care setting, and timely provision of comprehensive eye care (Quality of the evidence, low; Strength of the recommendation, strong).

**Recommendation 4:** Screening followed by provision of hearing aids should be offered to older people for timely identification and management of hearing loss (Quality of the evidence, low; Strength of the recommendation, strong).

***Prevent cognitive declines and depression, and promote psychological well-being***

**Recommendation 5:** Cognitive stimulation can be offered to older people with cognitive impairment, with or without a formal diagnosis of dementia (Quality of the evidence, low; Strength of the recommendation, conditional).

**Recommendation 6:** Older adults who are experiencing depressive symptoms can be offered brief, structured psychological interventions, in accordance with WHO mhGAP intervention guidelines, delivered by healthcare professionals with a good understanding of mental health care for older adults (Quality of the evidence, very low; Strength of the recommendation, conditional).

***Manage age-associated conditions such as urinary incontinence***

**Recommendation 7:** Prompted voiding for the management of urinary incontinence can be offered to older people with cognitive impairment (Quality of the evidence, very low; Strength of the recommendation, conditional).

**Recommendation 8:** Pelvic floor muscle training, alone or combined with bladder control strategies and self-monitoring, should be recommended for older women with urinary incontinence (urge, stress, or mixed) (Quality of the evidence, moderate; Strength of the recommendation, strong).

***Prevent falls***

**Recommendation 9:** Medication review and withdrawal (of unnecessary or harmful medication) can be recommended for older people at risk of falls (Quality of the evidence, low; Strength of the recommendation, conditional).

**Recommendation 10:** Multimodal exercise (balance, strength, flexibility, and functional training) should be recommended for older people at risk of falls (Quality of the evidence, moderate; Strength of the recommendation, strong).

**Recommendation 11:** Following a specialist’s assessment, home modifications to remove environmental hazards that could cause falls should be recommended for older people at risk of falls (Quality of the evidence, moderate; Strength of the recommendation, strong).

**Recommendation 12:** Multifactorial interventions integrating assessment with individually tailored interventions can be recommended to reduce the risk and incidence of falls among older people (Quality of the evidence, low; Strength of the recommendation, conditional).

***Support caregivers***

**Recommendation 13:** Psychological intervention, training, and support should be offered to family members and other informal caregivers of care-dependent older people, particularly but not exclusively when the need for care is complex and extensive and/or there is significant caregiver strain (Quality of the evidence, moderate; Strength of the recommendation, strong).

Providers are thus asked to ensure that (1) the assessment of individual impairments/declines in capacity is used to inform the development of a personalized care plan, and that all domains are assessed together; (2) interventions to improve nutrition and encourage physical exercise are included in most of the care plans and that all the interventions needed are delivered when possible in conjunction with each other; and (3) the presence of impairment or severe decline in capacity may require referral to a physician and/or specialist for comprehensive geriatric assessment, and/or diagnostic confirmation and treatment of a suspected associated disease (e.g., hypertension, diabetes, chronic obstructive pulmonary disease) should be given careful and, when appropriate, urgent consideration. WHO has developed clinical guidelines to address most of the relevant chronic diseases, and every healthcare provider should have access to these (S1 Text).

## Guideline implementation considerations

The recommendations in these guidelines need to be implemented using an integrated care approach following five steps listed below.

### Step 1: Assess older person’s needs and declining physical and mental capacities

Effective delivery of interventions recommended in this guideline should start with a comprehensive assessment of the intrinsic capacity of the older person; of the associated underlying conditions, behaviors, and risks that may influence future intrinsic capacity; and of the person’s physical and social environment. These assessments should include not only a traditional history-taking and, if appropriate, a physical examination but also a thorough analysis of the person’s values, priorities, and preferences concerning the course of their health and its management.

This assessment is essential to the development of a care plan and to tailoring interventions that are acceptable and appropriate for the older person. Another advantage is that it unites different providers around one goal: to maintain intrinsic capacity and functional ability. They can ensure that the necessary follow-up occurs and that links are made between health- and social care.

### Step 2: Define the goal of care and develop a care plan with multicomponent interventions

Integration of care can only be achieved if patients, services, and providers are working towards a single common goal: maintaining intrinsic capacity and functional ability [14]. It is essential that the older person is involved in decision-making and goal-setting from the outset, and that goals are set and prioritized in accordance with the person’s needs and preferences.

### Step 3: Implement the care plan using the principle of self-management support

This involves providing older people with the information, skills, and tools that they need to manage their health conditions, prevent complications, maximize their intrinsic capacity, and maintain their quality of life [15–17]. This does not imply that older people will be expected to “go it alone” or that unreasonable or excessive demands will be placed on them. It does, however, recognize their autonomy and abilities to direct their own care, in consultation and partnership with healthcare providers, their families, and a range of other carers. Routine healthcare visits provide excellent opportunities for providing self-management support. In this context, self-management support becomes an ongoing and integral part of clinical care [18–20].

### Step 4: Ensure a strong referral pathway and monitoring of the care plan

Regular and sustained follow-up of older people, with integration among different levels and types of care service, is essential for implementing the interventions recommended in these guidelines. Such an approach promotes early detection of complications or of changes in functional status, thus preventing unnecessary emergencies and related inefficiencies. It also provides a framework for monitoring progress against the care plan, as well as a means for arranging additional support as needed. Follow-up and support might be especially important following major changes in health status, treatment plan, or social role/situation (e.g., a change in residence or death of the partner).

### Step 5: Engage communities and support caregivers

Caregiving can be demanding, and caregivers of people with loss of capacity are often isolated and at high risk of experiencing psychological distress and depression. The guidelines include evidence-based recommendations to support caregivers. Beyond this, caregivers need basic information about the older person’s health conditions, and encouragement to develop a range of practical skills, such as how to transfer a person from a chair to a bed safely or how to help with bathing. The older person and/or caregiver should be provided with information about the community-based resources available to them. Opportunities to involve communities and neighborhoods more directly in supporting care must be explored, particularly by encouraging local volunteering and enabling older community members to contribute. The associations and groups that draw together older people are one mechanism by which such activities could happen.

## Limitations of the evidence and guidelines development process

Although transparent and robust methodologies were applied for evidence synthesis and the development of recommendations, these guidelines have some limitations.

One of the most challenging aspects of the process was generalizing the evidence to poorly resourced community healthcare settings. For example, all intervention trials for managing urinary incontinence (using prompted voiding techniques) came from high-income countries and studies were mainly conducted in residential or long-term care settings. The interventions were mainly implemented by nurse professionals involved in routine care. Although such studies provided evidence of efficacy in principle, the intensiveness of the intervention may be difficult to sustain in the community and in low- and middle-income countries, where primary caregivers of older people are likely to have other routine household and work responsibilities. In such instances, we took advantage of the GRADE methodology, which clearly recognizes that, in addition to the evidence base, other aspects that are expected to inform the recommendations include consideration of values such as gender equality, equity and the protection of human rights, feasibility and resource use, and the knowledge and experience of the GDG experts. The added value of GRADE in these circumstances is that it requires the GDG to transparently report that some recommendations are based on strong values but weak evidence [21]. These limitations emphasize the need to fill key knowledge gaps in ways that can enable improved future guidance.

Synthesizing evidence on the effects of complex interventions as judged by multiple outcomes can be challenging to conduct because it faces high diversity across studies and limitations in the data available. There are major implementation research questions that need to be addressed as a priority—these are listed in Box 2.

**Box 2. Key implementation research areas**

Although research in the field of older persons’ health and well-being has significantly advanced in recent years, most of these advancements have been driven by the needs of health systems in the richest countries. To appropriately translate research findings into clinical and public health practices, it is critical to accelerate implementation research to evaluate interventions beyond the controlled conditions of research settings and among populations that experience the largest losses of healthy life expectancy.

Research areas that will help in scaling up the coverage of these guidelines:

1. Strengthening human resources, including capacity development to promote healthy ageing and interventions to maintain capacity and ability at community level.
2. Improving health financing to avoid out-of-pocket and point-of-care payments for healthcare.
3. Simplifying interventions so that they can be delivered with fewer resources.
4. Identifying intervention delivery channels for greater reach and equity, and lower costs, including outreach into older persons’ homes and engagement of the nongovernment sector.
5. Improving program management and synergies between health- and social care services, and vertical programs such as eye care, to foster person-centered integrated care.
6. Engage communities to support delivery of interventions and increase healthcare utilization.
7. Scalable low-cost technologies for assessment and management of declines in intrinsic capacity of older adults.
8. Interventions to support caregivers at the community level.

Some examples of research questions:

1. What are the specific barriers that hinder access to effective high-value interventions described in the ICOPE guideline?
2. How can we use electronic and mobile technology to improve monitoring and follow-up of older people with steady declines in intrinsic capacity?
3. How can the coverage, quality, and impact of ICOPE recommendations be improved by involving community health workers and caregivers?
4. What is the cost to the health system of scaling up ICOPE recommendations when delivered through routine primary healthcare?
5. What is the effectiveness of eHealth applications targeting older people with complex care needs?

## Conclusion

In this article, we highlight the importance of evidence-based community-level interventions to manage declines in intrinsic capacity in older people, and supporting caregivers, using an integrated care approach. The guidelines are designed to help older people to maintain their physical and mental capacities and/or to slow or reverse any declines. They are community based and designed around the needs of the older person rather than the provider. They look to enable the effective coordination between health- and social care services and providers.

In the near future, further efforts should be made to introduce formal evaluations of the capability of these programs to induce relevant and persistent changes and to generate useful insights on how implementation in low-resourced healthcare settings should be conducted to maximize benefit at sustainable costs.

We hope that the publication of the guidelines and the underlying evidence base will stimulate interest in the global scientific community in strengthening evidence where it is weak and in innovating, for example, around interventions with broad application, using self-management with support of technologies. WHO will follow a systematic procedure for updating the recommendations following the same robust and transparent methodological process that was employed for their initial development. We also expect revisions to include a review of relevant new questions or areas currently not covered by the ICOPE guidelines.

Universal health coverage (UHC) is the foundation for achieving the health objective of the Sustainable Development Goals (SDGs). To achieve this objective, older people’s health- and social care needs must be addressed in an integrated manner and with continuity of care over the long term. The ICOPE recommendations can contribute to the SDGs and UHC goals and act as the basis for national guidelines and for the inclusion of Healthy Ageing interventions packages in primary care programs, using a person-centered and integrated approach.

## Supporting information

S1 Fig WHO ICOPE guidelines development process. ICOPE, Integrated Care for Older People; WHO, World Health Organization.
(DOCX)

S1 Text WHO methodology for the development of guidelines. WHO, World Health Organization.
(DOCX)

S2 Text WHO guidelines and resources related to ICOPE. ICOPE, Integrated Care for Older People; WHO, World Health Organization.
(DOCX)

## Abbreviations

GDG: guideline development group
GRADE: Grading of Recommendations Assessment, Development and Evaluation
ICOPE: Integrated Care for Older People
SDG: Sustainable Development Goal
UHC: universal health coverage
WHO: World Health Organization
