# Supplementary material for: Redesigning care for older people to preserve physical and mental capacity: WHO guidelines on community-level interventions in integrated care
Source: PLoS Med. 2019 Oct 18;16(10):e1002948. doi: 10.1371/journal.pmed.1002948 (PMC6799894; doi:10.1371/journal.pmed.1002948)
Supplement: S1 Fig — ICOPE, Integrated Care for Older People; WHO, World Health Organization. (DOCX) [file pmed.1002948.s001.docx]

**S1 Fig: WHO ICOPE (Integrated Care for Older People)** **guidelines development process**

**
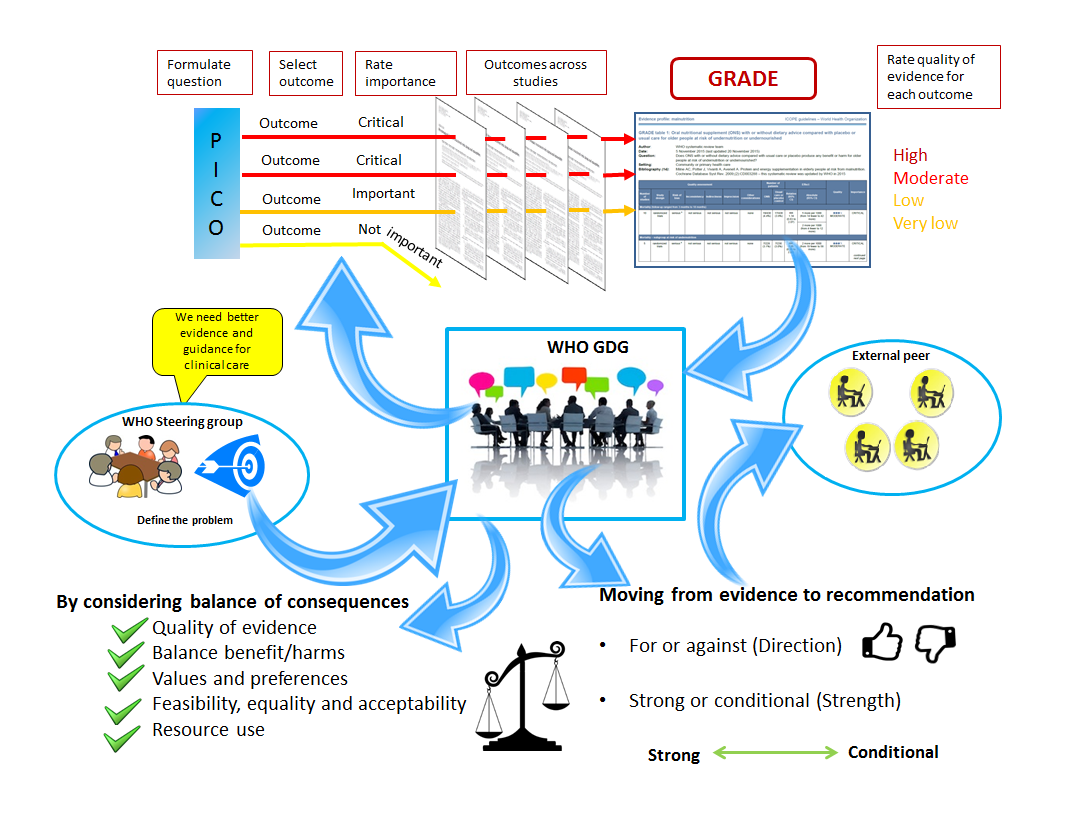
**

Source: WHO handbook for guideline development – 2nd ed. <http://www.who.int/publications/guidelines/handbook_2nd_ed.pdf>
