# Supplementary material for: Redesigning care for older people to preserve physical and mental capacity: WHO guidelines on community-level interventions in integrated care
Source: PLoS Med. 2019 Oct 18;16(10):e1002948. doi: 10.1371/journal.pmed.1002948 (PMC6799894; doi:10.1371/journal.pmed.1002948)
Supplement: S1 Text — WHO, World Health Organization. (DOCX) [file pmed.1002948.s002.docx]

**S1 Text: WHO methodology for the development of guidelines**

In summary, the WHO guideline steering group led by the Department of Ageing and Life Course was established with representatives from relevant WHO departments and programmes with an interest in providing scientific advice regarding the delivery of care to older people. Under the supervision of the steering group, two additional groups were formed: a guideline development group (GDG) and an external peer review group. The GDG included a panel of academics and clinicians with multidisciplinary expertise on the conditions covered by the guidelines, and geriatricians and doctors specialising in the care of older people. Consideration was given to the balance of gender and of geographical regions (including high-, middle- and low-income countries).

The scope of the guidelines and PICO questions (Population, Intervention, Comparison group, Outcomes) were predefined in consultation with the GDG and steering group (S1 Fig). Nine PICO questions were formulated. Outcomes were rated by GDG members and external experts according to the importance of each outcome from the perspectives of older people and service providers, as not important (rated 1–3), important (4–6), or critical (7–9). Outcomes rated as critical were selected for inclusion in the PICO analysis. When formulating the scoping questions and conducting the reviews, the focus was on evidence that applied specifically to older people who were frail or care dependent or had priority conditions, and on interventions that could be used by non-specialist health workers in community settings or primary health care. The information on management of conflict of interest and steps that were taken for evidence retrieval, assessment and synthesis are published elsewhere[[12](#_ENREF_12)]. The detailed evidence profile can be accessed from the WHO-ICOPE evidence resource centre (http://www.who.int/ageing/health-systems/icope/evidence-centre/en/).

In addition to the GDG members, four external peer reviewers provided independent expert input from specialized fields – psychiatry, nutrition, physical therapy and geriatric medicine. A preliminary version of the guidelines and the evidence profiles prepared by WHO staff and the GDG were circulated to the peer reviewers. Additionally, four external peer reviewers were asked to rate the quality of the guidelines using a slightly modified version of the tool, Appraisal of Guidelines for Research and Evaluation (AGREE II). The original AGREE II tool lists 23 key items in the following domains: scope and purpose, stakeholder involvement, rigour of development, clarity of presentation, applicability, and editorial independence[[16](#_ENREF_16)]. The reviewers’ total AGREE II scores ranged from 22 to 154, and the average was 122.2 The guidelines received the highest scores in six AGREE domains : a) scope and purpose ( 85%; b) stakeholder involvement (74%); c)rigorous of development (80%); c) clarity of presentation (83.3%); d) applicability (72.2%); e)editorial independence (95%). The overall methodological quality of the ICOPE guidelines was generally high (>70% in all six domains) in the independent appraisal using the AGREE II instrument.
