# Supplementary material for: Redesigning care for older people to preserve physical and mental capacity: WHO guidelines on community-level interventions in integrated care
Source: PLoS Med. 2019 Oct 18;16(10):e1002948. doi: 10.1371/journal.pmed.1002948 (PMC6799894; doi:10.1371/journal.pmed.1002948)
Supplement: S2 Text — ICOPE, Integrated Care for Older People; WHO, World Health Organization. (DOCX) [file pmed.1002948.s003.docx]

**S2 Text : WHO guidelines and resources related to ICOPE**

Mental Health Gap Action Programme (mhGAP)–mhGAP intervention guide for mental, neurological and substance use disorders in non-specialized health settings ,version 2.0 (2016): http://www.who.int/mental_health/mhgap/mhGAP_intervention_guide_02

Package of essential noncommunicable (PEN) disease interventions for primary health care in low-resource settings (2010): http://www.who.int/nmh/publications/essential_ncd_interventions_lr_settings.pdf

Guidelines for hearing aids and services for developing countries(2004): http://www.who.int/pbd/deafness/en/hearing_aid_guide_en.pdf

Global recommendations on physical activity for health(2010): <http://www.who.int/dietphysicalactivity/factsheet_recommendations>

WHO priority assistive products list (2016): <http://www.who.int/phi/implementation/assistive_technology/EMP_PHI_2016.01/en/>

| Mental Health Gap Action Programme (mhGAP)–mhGAP intervention guide for mental, neurological and substance use disorders in non-specialized health settings ,version 2.0 (2016): http://www.who.int/mental_health/mhgap/mhGAP_intervention_guide_02  Package of essential noncommunicable (PEN) disease interventions for primary health care in low-resource settings (2010): http://www.who.int/nmh/publications/essential_ncd_interventions_lr_settings.pdf  Guidelines for hearing aids and services for developing countries(2004): http://www.who.int/pbd/deafness/en/hearing_aid_guide_en.pdf  Global recommendations on physical activity for health(2010): <http://www.who.int/dietphysicalactivity/factsheet_recommendations>  WHO priority assistive products list (2016): <http://www.who.int/phi/implementation/assistive_technology/EMP_PHI_2016.01/en/> |
| --- |
